# Nicotine-induced Disturbances of Meiotic Maturation in Cultured Mouse Oocytes: Alterations of Spindle Integrity and Chromosome Alignment

**DOI:** 10.1186/1617-9625-2-16

**Published:** 2004-09-15

**Authors:** Maria Teresa Zenzes, Ryszard Bielecki

**Affiliations:** 1 Department of Obstetrics and Gynaecology, Division of Reproductive Sciences, University of Toronto, Toronto, Ontario, Canada; 2 Mount Sinai Hospital, 600 University Ave., Room 876, Toronto, Ontario M5G 1H5, Canada

## Abstract

We investigated whether nicotine exposure *in vitro *of mouse oocytes affects spindle and chromosome function during meiotic maturation (M-I and M-II). Oocytes in germinal vesicle (GV) stage were cultured in nicotine for 8 h or for 16 h, to assess effects in M-I and in metaphase II (M-II). The latter culture setting used the three protocols: 8 h nicotine then 8 h medium (8N + 8M); 16 h nicotine (16N); 8 h medium then 8 h nicotine (8M + 8N). Non-toxic concentrations of nicotine at 1.0, 2.5, 5.0 and 10.0 mmol/L were used. Spindle-chromosome configurations were analyzed with wide-field optical sectioning microscopy. In 8 h cultures, nicotine exposure resulted in dose-related increased proportions of M-I oocytes with defective spindle-chromosome configurations. A dose-related delayed entry into anaphase I was also detected. In 16 h cultures, nicotine exposure for the first 8 h (8N + 8M), or for 16 h (16N), resulted in dose- and time-related increased proportions of oocytes arrested in M-I (10 mmol/L; 8 h: 53.2%, controls 9.6%; 16 h: 87.6%, controls 8.5%). Defects in M-I spindles and chromosomes caused M-I arrest leading to dose-related decreased proportions of oocytes that reached metaphase-II (10 mmol/L 8 h: 46.8%, controls 90.4%;16 h: 12.4%, controls 91.5%). A delayed anaphase-I affected the normal timing of M-II, leading to abnormal oocytes with dispersed chromosomes, or with double spindles and no polar body. Nicotine exposure during the second 8 h (8M + 8N) resulted in dose-related, increased proportions of M-II oocytes with defective spindles and chromosomes (10 mmol/L: 42.9%, controls 2.0%). Nicotine has no adverse effects on GV break down, but induces spindle and chromosome defects compromising oocyte meiotic maturation and development.

## Introduction

Meiotic maturation of gametic cells (meiosis) is a process which, after two meiotic cell divisions, results in reduction to half (haploid) of the original (diploid) number of chromosomes. The meiotic spindle in oocytes is involved in these divisions. Its bipolar structure, established by acentriolar centrosomes which define spindle polarity and support microtubule nucleation, ensures that the replicated homologous chromosomes are segregated equally to the two daughter cells [1].

The meiotic spindle is useful for evaluating the impact of drugs and environmental toxins on oocyte meiosis and determining sensitive stages of meiosis [2]. The period of oogenesis preceding ovulation has been found to be very sensitive to chemically induced disturbances in meiosis [3-8].

A diverse group of chemicals and drugs to which humans are exposed have shown ability to interact with the microtubules of the meiotic spindle of mice and to disrupt its integrity [9-11]. Alkaloids such as colchicine and vinblastine are known to interact directly with the meiotic spindle apparatus without metabolic activation, leading to altered microtubular assembly and spindle function [12-15].

Nicotine, which accounts for about 95% of the total alkaloid content in cigarette tobacco [16] has shown to be genotoxic in mammalian cell systems *in vitro *[17,18]. Nicotine has adverse effects on reproduction, retarding embryonic growth and delaying embryo implantation in mouse and rats [19,20]. In hamster and mouse oocytes nicotine exposure *in vitro *induced meiotic blockage in metaphase-I [21,22], while administration *in vivo *led to a significant reduction in the number of ovulated oocytes in mice.

These studies analyzed the effects of nicotine on the chromosome status of oocytes during meiosis [21,22], but there are no studies on nicotine-induced disturbances to the integrity of the meiotic spindle. Because normal bipolar spindles have chromosomes aligned in the equatorial plate, the spindle assay allows morphological evaluations of deviations of normal spindle organization and chromosome alignment [2]. This method is sensitive as it detects even slight disruptions in spindle formation and chromosome alignment that could increase errors in chromosome segregation [23]. In the present study we performed a morphological evaluation of spindle morphological integrity and chromosome alignment in mouse oocytes exposed to nicotine *in vitro *during the first (M-I) and second (M-II) meiotic divisions. The spindle-disturbing action of nicotine was analyzed in relation to dose- and time-related effects of exposure.

## Animals and Methods

Female ICR (Institute of Cancer Research) mice (Harlan Sprague-Dawley; Indianapolis, IN, USA), 8–20 weeks of age (weight 25–34 g) were used. They were housed under a 12 h light:12 h dark photoperiod, in an ambient temperature of 21–23°C, and relative humidity of 50 ± 5%. Food and water were provided *ad libitum*. We did not use hormonal stimulation to avoid a possible confounding effect [3,9]. However, as each animal yielded about 40 oocytes without hormonal stimulation, a sufficient number of oocytes per experiment could be obtained for adequate statistical analysis.

### Preparation of oocytes

In each experiment, two females were killed by cervical dislocation. Ovaries were dissected out and were placed in M2 medium (Sigma, St. Louis, MO, USA). Under microscope surveillance, large antral follicles were then punctured to release cumulus-oocytes-complexes (COC) into M2 medium containing 1.4% BSA (Sigma). When released from the granulosa cells of the follicle, fully grown germinal vesicle-stage (GV) oocytes are capable of undergoing spontaneous maturation *in vitro *into metaphase II [3,24]. Oocytes were freed of granulosa cells by repeated pipetting and then transferred into a culture dish containing 100 μmol/L 3-isobutyl-I-methylxanthine/mL (3-IBMX; Sigma, I-7018) in 1 mL M16 medium (Sigma) to inhibit spontaneous oocyte maturation [24]. Oocytes were maintained in 3-IBMX at 37°C in an atmosphere of 5% CO_2 _until all oocytes were collected. After collection, oocytes were assessed under an inverted microscope and granular oocytes (about 5%) were discarded. Oocytes were then washed three times in M16 medium and incubated in M16 medium at 37°C, 5% CO_2_, for defined times in the presence or absence of nicotine.

### Preparation of nicotine and culture settings

For each experiment a stock solution of nicotine (lot 29H0467; Sigma N-5260) of 20.0 mmol/L was freshly prepared in M16 culture medium. The final concentrations of nicotine were adjusted in M16 medium, and the pH adjusted to 7.5 within 1 hour prior to placing oocytes in culture.

A cytogenetic investigation that tested an effective non-cytotoxic concentration of nicotine using a range of concentrations (1.0, 5.0, 10.0 and 20.0 mmol/L) found that the maximum non-toxic dose was 10.0 mmol/L [22]. Here we confirmed a non-cytotoxic effect of nicotine at 10.0 mmol/L with the trypan blue exclusion method. For this, 20 GV oocytes were exposed *in vitro *to 10.0 mmol/L of nicotine, or only medium, and were then stained with 0.4% trypan blue (Sigma) and assessed 5 min, 10 min, and 2 h later for cytoplasmic staining by light microscopy. As a positive control, 10 granular GV oocytes were stained and assessed. None of the treated and control oocytes took up the stain at these three times, while the granular oocytes did so.

### Culture settings

To assess nicotine effects on the rate of GV-to-M-I transition, GV oocytes were cultured for eight hours in nicotine concentrations of 1.0, 2.5, 5.0, and 10.0 mmol/L, or in medium only, and were fixed. To analyze nicotine effects on GV to MI-MII transition and time-effect of exposure, GV oocytes were cultured for 16 h using three different protocols: 8 h in nicotine followed by 8 h in M-16 medium (8N + 8M), 8 h in medium followed by 8 h nicotine (8M + 8N), and 16 h in nicotine (16N). Control GV oocytes were cultured for 8 h + 8 h or for 16 h in M-16 medium only. These experimental protocols were replicated until adequate numbers of oocytes (≥ 150) for each nicotine concentration were obtained for analysis.

### Immunofluorescence

At the end of culture (8 h, or 16 h), oocytes were fixed for 20 min in a microtubule-stabilizing buffer containing 2.0% formaldehyde, 0.5% Triton X-100, and 1 μmol/L Taxol (Sigma). Oocytes were then washed three times in a blocking solution of PBS containing 2% bovine serum albumin (BSA), 2% Carnation powdered skim milk, 2% normal goat serum, 0.1 M glycine, and 0.01% Triton X-100 (Sigma). Oocytes were then stored up to three days in this solution at 4°C.

Immunostaining of oocytes was performed according to the method described in Baka et al. [25] with some modifications. Briefly, oocytes were treated with monoclonal mouse antibody to α-tubulin (clone DM-IA; ICN Biomedical Inc., Costa Mesa, CA, USA) for 1 h at 37°C, diluted 1:100 in a washing solution (PBS containing 0.1% BSA and 0.02% sodium azide). Oocytes were then treated for 1 h in blocking solution and further incubated in fluorescein-conjugated goat anti-mouse immunoglobulin (IgG; ICN Biomedicals Inc.) diluted 1:200 in washing solution for 1 h at room temperature.

After that, oocytes were soaked in washing solution and stained with 4',6',-diamidino-2-phenylindole (DAPI; Sigma) to stain chromosomes. After a brief wash in PBS, oocytes were mounted on slides in 90% glycerol in PBS containing 100 mg/mL of DABCO (Sigma) as an anti-fading agent.

### Microphotography

Microscopic preparations of oocytes were analyzed in a Zeiss Axioplan Photomicroscope (Jena, Germany) equipped with epifluorescent UV light and corresponding excitation and barrier filters. Selected microscopic preparations were imaged on an O2 Silicon Graphics Work Station with a DeltaVision^® ^Deconvoluted Microscope (Applied Precision Inc., Issaquah, WA, USA), a wide-field optical sectioning microscope. The figures were then composed using Adobe Photoshop 5.0 Education Version on a computer. The random orientation of the spindle yielded photographs in which the equatorial plane of the spindle was positioned at any angle up to 90° (where the spindle is being seen from a pole).

### Statistical analysis

Standard statistical packages (StatView, SAS Institute, Cary, NC, USA and SPSS, Inc., Chicago, IL, USA) were used for recording and analyzing data. Statistical analyses were done by contingency chi-square (χ^2^) for comparing proportions, and by weighted regression for dose-response, weighting each proportion of oocytes with abnormal spindle-chromosomes by its corresponding total number of oocytes [26].

## Results

### Controls

During *in vitro *culture of mouse GV oocytes, germinal vesicle breakdown (GVBD) is completed in two hours; prometaphase predominates after 6 to 8 h of culture, metaphase-I at 10 to 14 h, and a majority of oocytes reach metaphase-II at 16 h of culture [27]. Similarly, in our study, after 8 h of culture a large majority (361/394: 91.6%) of control GV oocytes have achieved prometaphase/metaphase-I, and 8.4% were in anaphase-I/telophase-I (Table 1). After 16 h of culture a majority (91.5%) of control oocytes were in metaphase-II with an extruded polar body.

**Table 1:** Effects of nicotine on GV to M-I transition. Numbers and (%).

**8N Treatment (8 h in Nicotine)**											
				**Abnormal Pro/metaphase-I**						**Normal Metaphase-I**	**Normal Anaphase-I**
**Nicotine mmol/L**	**Total**	**GV**^a^	**N**^b^	**No**	**Apo**	**Trip**	**Diso**	**Disp**	**Total**		

0.0	494	100	394	4	2	3	3	5	17	344	33
		(20.2)							(4.3)	(87.3)	(8.4)
1.0	262	45	217	0	4	0	0	12	16	181	20
		(17.2)							(7.4)	(83.4)	(9.2)
2.5	239	57	182	0	0	2	0	12	14	156	12
		(23.8)							(7.7)	(85.7)	(6.6)
5.0	342	69	273	3	7	7	8	14	39	230	4
		(20.2)							(14.3)	(84.2)	(1.5)
10.0	308	66	242	26	9	13	19	11	78^c^	164^d^	0^e^
		(21.4)							(32.2)	(67.8)	(0.0)

Abnormal Pro/metaphase Configurations: No: no spindles; Apo: apolar; Trip: tripolar; Diso: disorganized spindles; Disp: displaced chromosomes.

^a^Oocytes in germinal vesicle (GV) stage.

^b^Total number of oocytes – number of GV oocytes.

^c^Dose-related effect (pooled data vs. control) (p = .001).

^d^p = .009 only at 10 mmol/L.

^e^Dose-related effect (p < .0001)

### Effects of nicotine on GV to MI transition (8N)

No effect of culture conditions or nicotine exposure and dose was detectable on GV to MI transition. Using phase contrast microscopy, similar proportions (about 20%) of GV oocytes were found both in control and nicotine-treated oocytes (Table 1). The proportions of normal M-I oocytes are similar to controls at nicotine concentrations of 1.0, 2.5 and 5,0 mmol/L, but at 10 mmol/L a reduction was detectable. The proportions of M-I oocytes with defective spindles increased with increasing nicotine dose, while the proportions of ana-phase-telophase I oocytes decreased in dose-related manner, with total inhibition at 10 mmol/L (Table 1).

### Dose- and time-related effects of nicotine exposure (8N + 8M; 16N)

**Figure 1:**
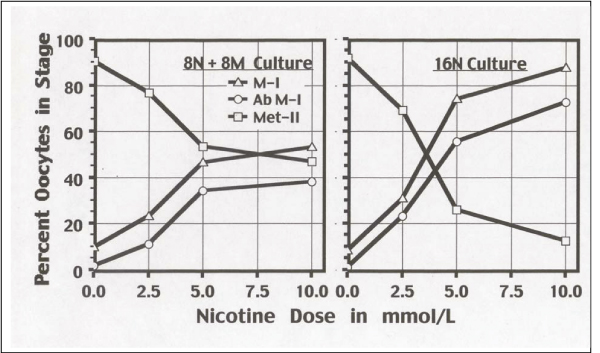
**Percent Oocytes in Meiosis Stage According to Nicotine Treatment**. 8N + 8M culture is 8 h in nicotine, then 8 h in medium. 16N culture is 16 h in nicotine. Percent oocytes in M-1, abnormal M-I, and meta-phase II are shown. Abnormal M-I are included in M-I.

**Figure 2:**
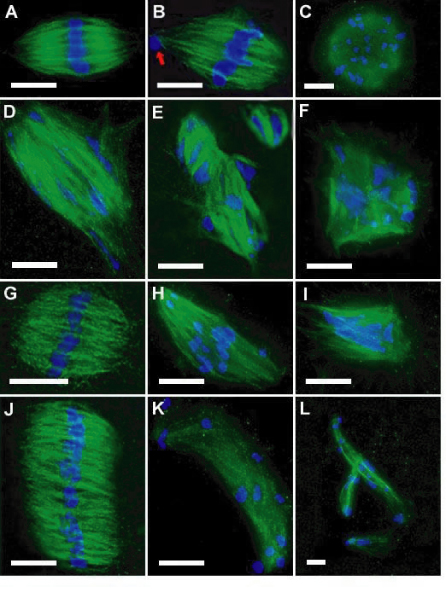
**Abnormal Spindle-Chromosome Configurations in M-I and M-II oocytes**. A-F: metaphase-I oocytes; G-L: metaphase-II oocytes (spindles are green and chromosomes are blue). Normal spindle (A); bipolar spindle with a displaced chromosome in the pole (B); apolar spindle (C); disorganized spindle (D); multiple spindles (E); multipolar spindle (F); normal metaphase-II spindle (G); bipolar spindle with displaced chromosomes near the poles (H); tripolar spindle (I); diploid (merged) spindle (J); very elongated spindle (K); multiple elongated spindles (L). Bar = 10 μm.

In 16 h cultures, nicotine exposure during the first 8 h (8N + 8M), or after continuous exposure for 16 h (16N) resulted in a dose-related blockage at M-I (pro-metaphase-metaphase I; Figure 1). The M-I dose-related arrest included both normal-looking oocytes (e.g., normal bipolar spindles and chromosomes aligned in the equatorial plane; Figure 2A) and abnormal oocytes either with absent spindles (not shown) or with various aberrant types of spindles-chromosomes configurations (Figure 2B–G). The block in M-I resulted in a dose-related reduction of oocytes that reached metaphase II (Figure 1). Comparisons between both culture settings (8N + 8M vs. 16N) showed a time-related effect on the proportions of oocytes blocked in M-I, and also on the proportion of oocytes that reached metaphase-II with a PB (p < .0001).

### Effects of nicotine on M-I to M-II transition (8M + 8N)

When nicotine was added in the second 8 h period, reduction in the proportion of normal metaphase II oocytes was found at 10 mmol/L, with no effect at lower concentrations (see Table 2). A positive dose-related response (p < .0001) in the proportion of abnormal metaphase II oocytes with aberrant spindle configurations (Figure 2H–L), or with normal bipolar spindles but with unaligned chromosomes (Figure 2K) is also shown in Table 2.

**Table 2:** Effects of nicotine on M-I to M-II transition. Numbers and (%).

**8M + 8N Treatment (8 h in medium, then 8 h in nicotine)**								
**Nicotine Mmol/L**	**Total**	**GV**	**N**^a^	**M-I**	**M-II**	**M-II Normal**	**M-II Abnormal**	
							
							**Aberrant**^b^	**Unaligned**^c^

0.0	230	61	169	17	152	148	1	3
		(26.5)		(10.1)	(89.9)	(87.6)	(0.6)	(1.8)
1.0	136	11	125	13	112	91	7	18
		(8.1)		(10.4)	(89.6)	(72.8)	(5.6)	(11.2)
2.5	255	69	186	23	163	145	14	4
		(27.0)		(12.4)	(87.6)	(78.0)	(7.5)	(2.2)
5.0	226	59	167	23	144	124	10	10
		(26.1)		(13.8)	(86.2)	(74.3)	(6.0)	(6.0)
10.0	258	36	222	54	168	94	45	29
		(14.0)		(24.3)	(75.7)	(42.3)^d^	(20.3)^d^	(13.1)^d^

^a^N = Total number of oocytes minus GV oocytes

^b^Aberrant spindles: tri- or multipolar, disorganized, double, multiple, or with a large polar body.

^c^Normal bipolar spindles but with chromosomes not aligned at the equator.

^d^Dose-related effect at 10.0 mmol/L (p = .0001)

### Effects of nicotine on diploid oocytes

In all 16 h culture settings (8N + 8M, 16N and 8M + 8N) we also found oocytes with no PB showing either two single spindles, or a double spindle (e.g., as wide as two single spindles) with an apparent double set of chromosomes aligned in the equatorial plate (Figure 2J). Data are shown in Table 3. The proportions of such abnormal diploid oocytes were higher than in controls at 2.5 mmol/L (p < .0001), but not at higher concentrations. The weighted regression using 1.0 mmol/L concentration (assessed in 8M + 8N cultures) confirmed a very significant (p < .0001) dose effect, with a peak at 2.5 mmol/L. A time-related effect at 2.5 mmol/L (8N + 8M vs. 16 N, p < .0001) was also found (Table 3).

**Table 3:** Effects of nicotine on diploid oocytes with three treatments. Numbers and (%).

	**8N + 8M**		**16N**		**8M + 8N**	
			
**Nicotine mmol/L**	**N**	**Diploid**^a^	**N**	**Diploid**^a^	**N**	**Diploid**^a^
0.0	157	0	261	1	169	1
		(0.0)		(0.4)		(0.6)
1.0		n.a.^b^		n.a.^b^	125	3
						(2.4)^c^
2.5	190	6	195	63	186	10
		(3.2)^d^		(32.3)^e^		(5.4)^f^
5.0	131	1	229	15	167	1
		(0.6)		(6.6)		(0.6)
10.0	201	1	259	5	222	2
		(0.5)		(1.9)		(0.9)

N = Total number of oocytes minus GV oocytes.

^a^Diploid = Oocytes with no polar body but with two single spindles or a double spindle with a double set of chromosomes.

^b^Not assessed with this treatment.

^c^Dose-effect confirmed using this concentration in this setting (p < .0001); a peak is at 2.5 mmol/L.

^d, e, f^Peak at 2.5 mmol/L (p < .0001).

^d vs. e^Time-related effect (p < .0001).

### Abnormal spindle-chromosome configurations

Figure 2 depicts pictures of defective oocytes arrested in metaphase-I (Figure 2B–F) and in metaphase-II (Figure 2H–L). For comparison normal bipolar spindles in metaphase I (Figure 2A) and in metaphase II (Figure 2G) are also shown. The different types of aberrant spindle-chromosome configurations include: i) M-I oocytes with no spindles, only chromosomes arranged in a bundle (not shown); ii) M-I spindles showing no evident polarity (Figure 2C); iii) M-I and M-II bipolar spindles with ≥ 1 chromosomes displaced to one or both spindle poles (Figure 2B, H), or with chromosomes not aligned in the equator but dispersed along the spindle axis (Figure 2D, K); iv) M-I spindles showing severely damaged microtubules with one or more chromosomes outside the spindle boundaries (Figure 2D, E, K); v) M-I oocytes with no PB showing two single bipolar spindles, or a double spindle with a double set of chromosomes aligned in the spindle equatorial plate (Figure 2J); vi) M-I and M-II oocytes with multiple (≥ 3) spindles of different sizes with dispersed chromosomes (Figure 2E, L); vii) M-I and M-II tripolar or multi-polar spindles with chromosomes dispersed or in discrete groups (Figure 2F, I); and viii) metaphase-II oocytes with a very large polar body (not shown).

## Discussion

The present study was designed to evaluate effects of nicotine exposure *in vitro *on the meiotic maturation of mouse oocytes. This information has relevance to humans because cigarette smoking, a prevalent hazardous habit, is known to adversely affect the reproductive health of women. Maternal smoking causes delay in conception [28], and is considered a risk factor for spontaneous abortion [28,29] and for trisomy 21 in offspring [30]. It is also associated with significant reductions in the number of ovulated oocytes [28,29,31-34]. With advancing age, the decline in oocytes is faster in smokers than in non-smokers [31,35], and leads to a significant reduction of the age of natural menopause in smokers [36], a clinical manifestation of follicle and oocyte depletion. These findings show that human oocytes are very sensitive to chemicals contained in cigarette smoke, and emphasize the need for experimental animal data to understand the underlying causes of meiotic perturbation.

In the present study, nicotine had no effect on germinal vesicle (GV) breakdown but induced blockage at M-I. Using chromosomal analysis of hamster oocytes exposed *in vitro *to 5.0 mmol/L of nicotine, Racowsky and colleagues [21] also found no effects on GV breakdown, and a block in M-I. In our present study the morphological analysis of fluorescent spindles permitted the recognition of two underlying causes for M-I arrest: inhibition of spindle formation, and severe damage to the spindle structure affecting chromosome alignment and segregation. The presence of M-I arrested oocytes with absent spindles or apolar spindles indicates that this damage must have occurred after germinal vesicle breakdown, when the chromosomes initiate spindle nucleating activity [37,38].

A prolonged M-I arrest resulted in delayed ana-phase-I entry, as indicated by significant dose-related reductions in the proportion of anaphase-I oocytes, with total inhibition at 10 mmol/L. Using *in vitro *exposure of hamster oocytes to nicotine at 5.0 mmol/L, Racowsky and colleagues [21] found reduced rates of ana-phase I oocytes with non-disjoined bivalents. In the present study, a delayed anaphase-I entry influenced the normal timing into metaphase-II; this is recognized in dose-related proportions of oocytes showing bipolar spindles but with chromosomes dispersed along the spindle, indicating delay to congress to the spindle's equatorial plate. Chromosome congression is the process of alignment of chromosomes at the spindle equator, and is mediated by kinetochores [39-44]. Whether this delay should affect the second meiotic division needs to be analyzed after fertilization.

The formation of severely defective spindles from nicotine exposure during M-I and M-II indicates a dynamically unstable system of microtubule damage that upsets the equilibrium of tubulin polymerization-depolymerization (e.g., spindle assembly and disassembly), thus altering chromosome alignment. Oocytes with unaligned chromosomes occur from failure of kinetochore anchorage to the microtubules during spindle formation [40], and are also found in spindle preparations of human oocytes exposed to low temperature [23]. Oocytes with multiple spindles occur because individual chromosomes can organize individual bipolar spindles that subsequently coalesce [37]. The formation of tripolar and multipolar spindles with defects in chromosome congression arise possibly from failure of individual chromosomes to form stable bipolar attachments. Metaphase-II oocytes with a very large polar body can also be induced by other chemicals and are classified as equally divided [6,7]. The formation of such an array of defective spindles compromises the viability and developmental potential of maturing oocytes. Indeed, in female mice treated *in vivo *with an acute dose of 10 mmol/L of nicotine, a reduction of 50% of ovulated oocytes was found [22]. In women using assisted conception a reduction of ovulated oocytes of about 17% was estimated in heavy smokers of ≥ 10 cigarettes/day [29].

Studies on the effects of other alkaloids on oocyte meiosis using chromosome analysis found comparable results. Vinblastine sulfate administered *in vivo *into female mice induced dose-related increases in the proportion of oocytes arrested in M-I; this was caused by inhibition of spindle microtubules, resulting in blockage in M-I [45-47]. In these studies diploid oocytes which have not yet formed a polar body were found; our study confirms a dose-response for oocyte diploidy from nicotine. Colchicine and cocaine were also found to induce inhibition of the spindle apparatus and alterations of microtubular assembly [48-50], as we found for nicotine in dose-related manner in this study.

Diploid M-I oocytes had two spindles or a double spindle with no first polar body extruded. Double spindles were centrally located (Figure 1J) suggesting a nicotine effect that inhibits migration of the spindle to the cortex. Using chromosome analysis, a similar behavior for chemically-induced diploid oocytes was observed [51], namely two spatially separated haploid spindles that eventually merge, assembling into a common spindle. Oocyte diploidy dramatically impairs the development of zygotes and embryos [52]. A cytogenetic investigation of oocytes from women in assisted conception who smoked ≥ 10 cigarettes/day found increased numbers of oocytes with diploid complements of 46 chromosomes, and of triploid zygotes with 69 chromosomes (produced from digynic fertilization) in dose-related association with number of cigarettes smoked per day [33]. In the present study, the threshold level for diploidy induction was clearly detectable at 1.0 mmol/L, and may even be lower. In women the serum nicotine concentration of heavy smokers (≥ 30 cigarettes day) was measured to be 23.7 ± 10.3 μg/L [53]. This demonstrates that the threshold level for diploidy induction is much lower in women smokers than in mice (1.0 mmol/L = 0.162 mg/mL), but this is over many years of daily nicotine exposure.

In conclusion, our study confirms that nicotine, the major alkaloid in cigarette tobacco, affects meiotic spindle function; it interferes with first meiotic cycle progression and upsets the temporal biochemical process of meiotic maturation, also found in hamster oocytes [21], and in women in assisted conception who smoke [29,33,34]. The present results are also consistent with a number of animal studies which demonstrate that oocyte exposure to various chemicals and drugs prevents or delays onset of anaphase I [3-5]. Perturbations in the temporal sequence of oocyte meiosis compromise the developmental competence of ovulated oocytes, with adverse consequences for reproduction.

### Previous Presentation

Presented in part at the 47th Annual Meeting of the Canadian Fertility and Andrology Society, Whistler, B.C., Canada, October 3–6, 2001, and at the 1st Annual Meeting of the International Society for The Prevention of Tobacco Induced Diseases, Essen, Germany, October, 28–29, 2002.

## Competing interests

The authors declare that they have no competing interests.
